# Plasmacytoma in patients with multiple myeloma: morphology and immunohistochemistry

**DOI:** 10.1186/s12885-020-06870-w

**Published:** 2020-04-22

**Authors:** Maiia V. Firsova, Larisa P. Mendeleeva, Alla M. Kovrigina, Maxim V. Solovev, Valery G. Savchenko

**Affiliations:** 1 Department of High-Dose Chemotherapy of Paraproteinemic Hemoblastoses, FSFI «National Research Center for Hematology», 4a Novyi Zykovskii pr, Moscow, Russian Federation 125167; 2 Department of Pathology, FSFI «National Research Center for Hematology», Moscow, Russian Federation; 3 FSFI «National Research Center for Hematology», Moscow, Russian Federation

**Keywords:** Multiple myeloma, Plasmacytoma, Extramedullary disease, CD 166, Ki-67, CD56, CXCR4, C-MYC, Immunohistochemistry

## Abstract

**Background:**

To study the histological structure and immunohistochemical (IHC) parameters of the plasmacytoma tumour substrate in patients with multiple myeloma (MM).

**Methods:**

The study included 21 patients (10 men/11 women) aged 23 to 73 years old with newly diagnosed MM complicated by plasmacytoma. Bone plasmacytoma was diagnosed in 14 patients, and extramedullary plasmacytoma was diagnosed in 7 patients. Plasmacytoma tissue specimens were examined using a LEICA DM4000B microscope. Anti-CD56, anti-CD166, anti-CXCR4, anti-Ki-67, and anti-c-MYC antibodies were used for IHC study of plasmacytoma biopsies.

**Results:**

When comparing the morphology of bone and extramedullary plasmacytoma, no significant differences were revealed; however, the substrate of extramedullary plasmacytoma was more often represented by tumour cells with an immature morphology than was the bone plasmacytoma substrate (57.1% vs. 28.6%, respectively). We revealed a significant difference in the expression of CD166 between bone and extramedullary plasmacytoma. The mean values ​​of CD166 expression in bone plasmacytoma cells were significantly higher (36.29 ± 7.61% versus 9.57 ± 8.46%, respectively; *p* = 0.033) than those in extramedullary plasmacytoma cells. We noticed that in extramedullary plasmacytoma cells, there were higher values for the Ki-67 index than in bone plasmacytoma cells, and this result was independent of cell morphology.

**Conclusion:**

The mechanisms involved in the dissemination of tumour plasma cells are currently unexplored. Even in such a small sample, some differences in expression could be identified, which may indicate that different mechanisms lead to the formation of bone and extramedullary plasmacytomas. Specifically, the expression of CD166 in extramedullary plasmacytoma cells was almost 4 times lower than that in bone plasmacytoma cells, which may indicate the involvement of CD166 in the mechanisms of bone destruction. The proliferative activity of extramedullary plasmacytoma cells was shown to be higher than that of bone plasmacytoma cells.

## Introduction

The introduction of highly sensitive imaging methods into clinical practice has facilitated the more frequent detection of bone and extramedullary plasmacytoma in patients with multiple myeloma (MM). In the case of intraosseous tumour growth, destruction of the cortical bone occurs, and the plasmacytoma extends beyond the bone borders, invading the surrounding tissues or spinal canal. In the case of haematogenous dissemination of plasma cells, isolated extramedullary plasmacytomas are formed in various organs and tissues without anatomical association with the bone. Invasive surgical procedures can lead to local plasmacytoma formation in the area of manipulation [1–3]. According to the results of clinical studies, it has been revealed that in patients with newly diagnosed MM, the occurrence of plasmacytoma varies from 3.5 to 18%, and in patients with relapse of the disease, it varies from 6 to 30% [1, 3–8]. The variability in these values is due to the lack of clear terminology-defining criteria. Thus, some authors include both bone plasmacytoma and extramedullary plasmacytoma under the concept of ‘extramedullary lesion’, while other researchers exclude bone plasmacytoma from the spectrum of extramedullary myeloma [1, 3, 5, 6]. If we define the term “extramedullary lesion” to consider only haematogenously formed plasmacytomas not related to the bone, the frequency of detection of such plasmacytomas in newly diagnosed MM does not exceed 3.5–6%. In published papers, insufficient attention has been paid to the study of the plasmacytoma tumour substrate morphological structure in patients with MM. In a small series of observations, it was shown that bone plasmacytoma cells are characterized by a mature morphology and that the substrate of extramedullary plasmacytoma is more often represented by cells with an immature morphology [3, 9–12].

The reasons why myeloma cells “escape” bone marrow stromal microenvironment control, yielding the formation of bone and extramedullary plasmacytomas, are being actively studied. The pathogenesis of extramedullary tumour growth is complex, has not been studied in detail and is probably due to loss of the interaction between plasma cells and the bone marrow microenvironment, which plays crucial roles in the processes of cell proliferation and migration. Among the causes of extramedullary plasma cell relocation, downregulation of adhesion molecule (CD56 and VLA-4) expression, dysregulation of chemokine receptors (CCR1, CCR2, and CXCR4), and an increase in heparanase-1 activity are suggested [1, 3]. These mechanisms are currently only suggested, since there are no scientific studies on a large number of patients, and the results of studies of small patient populations presented in the literature are contradictory.

Thus, it is important to assess the morphological patterns of bone plasmacytoma and extramedullary plasmacytoma biopsies and analyse the expression of a series of markers that presumably participate in the pathogenesis of myeloma cells spreading beyond the bone marrow.

**The background** of the work was to study the histological structure and IHC parameters of the plasmacytoma tumour substrate in patients with MM.

## Methods

The study included 21 patients (10 men and 11 women) aged 23 to 73 years old with newly diagnosed MM complicated by plasmacytoma. Patients with a solitary plasmacytoma were excluded. Bone plasmacytoma was diagnosed in 14 patients, and extramedullary plasmacytoma was diagnosed in 7 patients. In 3 out of the 7 patients with extramedullary plasmacytoma, bone plasmacytoma was also simultaneously diagnosed, but only extramedullary plasmacytoma was histologically confirmed. Bone plasmacytoma was relatively often found in the flat bones - the vertebrae, ribs, and skull bones. Isolated cases of clavicle or humerus lesions were recorded. Extramedullary plasmacytoma was found in various organs and tissues, including the liver, stomach, abdominal cavity and retroperitoneal space and in the soft tissues of the neck, breast, muscles of the shoulder girdle, and skin.

MM diagnosis was verified in accordance with the criteria developed by the International Multiple Myeloma Working Group 2014. Diagnostic methods included bone marrow cytological examination, immunochemical blood and urine examination, X-ray examination of the skeletal bones, and clinical blood and blood chemistry analyses. The characteristics of patients including the immunochemical variant of myeloma, Durie-Salmon stage, ISS stage, LDH, Ca^2+^ and haemoglobin values are presented in Table 1. The follow-up period ranged from 5 to 62 months (median: 24 months).

**Table 1:** Patients characteristics at diagnosis

Parameters	MM patients (***n*** = 21)
Median age, years (range)	55,9 (23–73)
Gender	
male / female	10/11
Plasmacytoma	
Bone	14
Extramedullary	7
Multiple myeloma subtype	
IgG	11
IgA	5
Light chain only	4
IgA + IgG	1
Durie-Salmon Stage	
IIA	8
IIIA	13
International Staging System stage	
I	8
II	9
III	4
Median Ca^2+^, mmol/l (range)	2,5 (1,39-2,85)
Median LDH, U/l (range)	370,5 (167–766)
Median Hemoglobin, g/l (range)	121 (86–156)

In all patients, plasmacytoma biopsy samples were collected after written informed consent was given by each person, according to the guidelines of local ethical committees and the Helsinki Declaration. This study was approved by the Local Ethical Committee of FSFI «National Research Center for Hematology» of the Ministry of Healthcare of the Russian Federation.

Plasmacytoma biopsy was performed on all patients before treatment. The plasmacytoma tissue specimens were examined using a LEICA DM4000B microscope. An anti-CD56, anti-CD166, anti-CXCR4, anti-Ki-67, and anti-c-MYC antibodies were used for the IHC study of the plasmacytoma biopsy specimens. Ten fields of view were viewed at 400-fold magnification to determine protein expression. The evaluation of marker expression was carried out using a semiquantitative method. The percentage of cells expressing a protein was calculated relative to the total number of cells in the tumour substrate. Where staining with antibodies was observed, the following values of expression were considered positive: ≥ 10% for CD56, ≥ 50% for CD166, ≥ 20% for CXCR4, and ≥ 40% for c-MYC [13–17]. The level of proliferative activity is expressed as a percentage and was assessed based on the expression of the Ki-67 marker by tumour cells.

Frequency analysis (cross tables, the Fisher-Freeman test) was used for statistical analysis. Student’s t-test was used to assess the significance of differences between two independent samples*.* The critical level of significance, ‘p’, was set as 0.05. Calculations were carried out using SPSS 16.0.2 statistical software.

## Results

### Histological examination of plasmacytoma in MM patients

Depending on the cell composition, 2 morphological variants were distinguished in the plasmacytoma biopsy specimens:

Variant I: massive infiltration by mature plasma cells; and.

Variant II: massive infiltration by plasma cells with an immature morphology (more than 50% of the tumour cells in the specimen were proplasmocytes and more than 5% of the tumour cells were plasmoblasts).

**Fig. 1:**
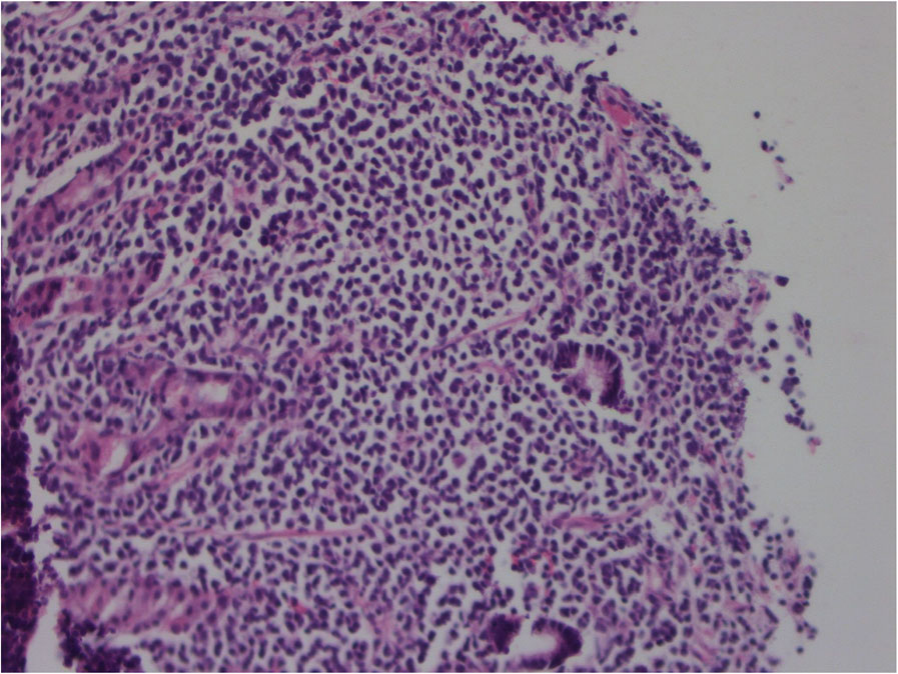
The histology of stomach extramedullary plasmacytoma in MM patients. Hematoxylin + eosin staining. Massive infiltration by mature plasma cells, × 200

**Fig. 2:**
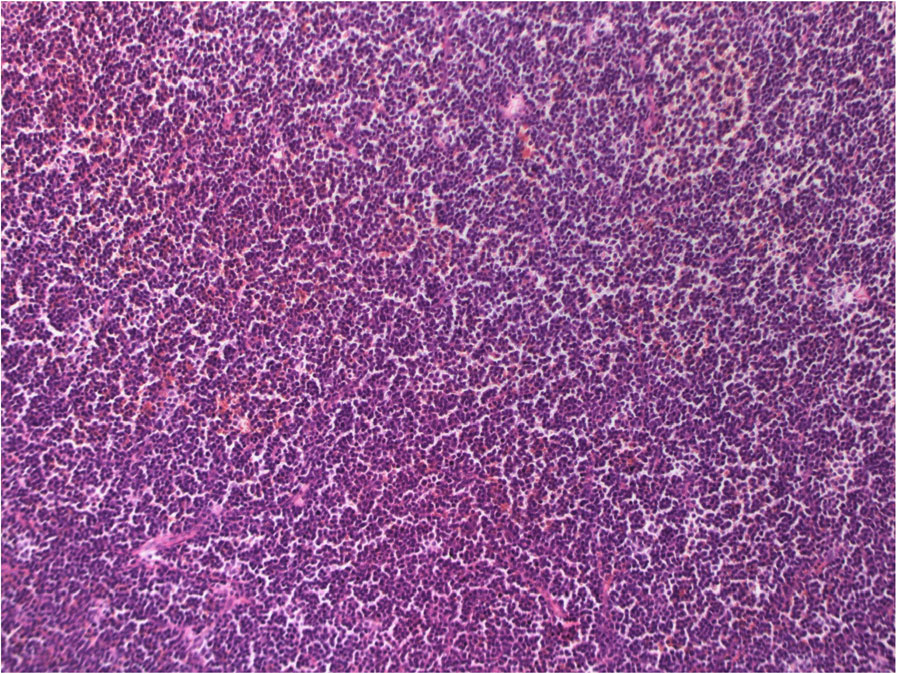
The histology of rib bone plasmacytoma in MM patients. Hematoxylin + eosin staining. Massive infiltration by mature plasma cells, × 200

**Fig. 3:**
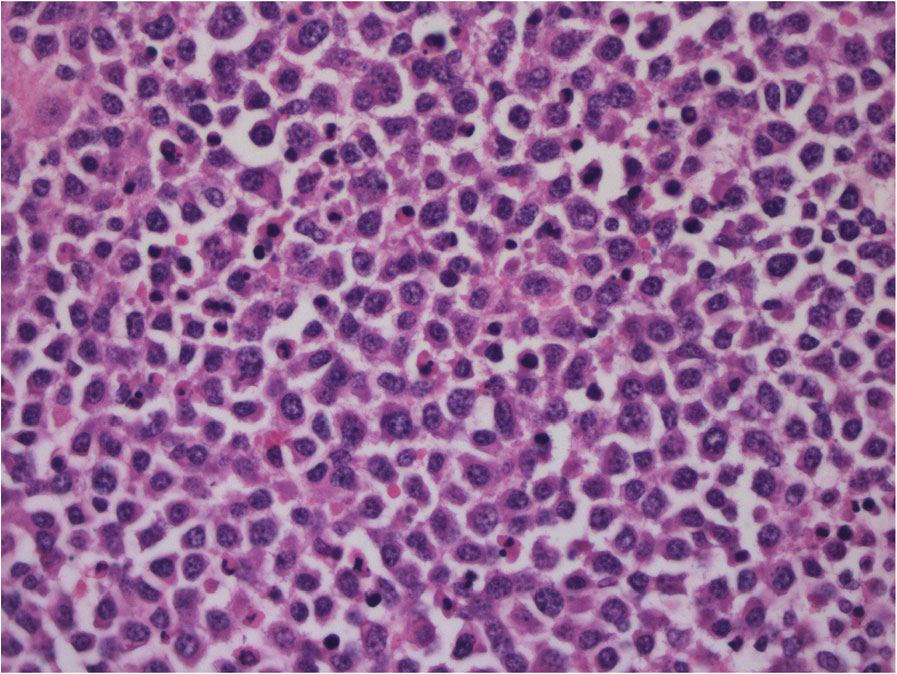
The histology of skull bone plasmacytoma in MM patients. Hematoxylin + eosin staining. Massive infiltration by tumor cells with immature morphology (plasmablasts have the blast structure of the nucleus, large nucleoli, and narrow cytoplasm);. × 400

**Fig. 4:**
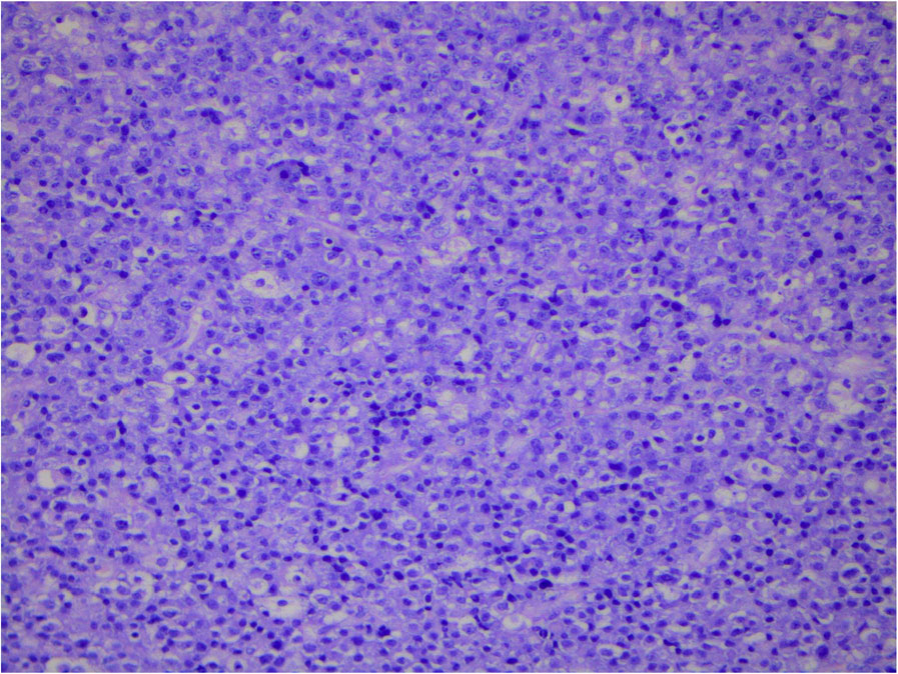
The histology of extramedullary plasmacytoma in the soft tissues of the neck in MM patients. Hematoxylin + eosin staining. Massive infiltration by tumor cells with immature morphology, × 200

Figures 1, 2, 3 and 4 present images of plasmacytoma tissue specimens that illustrate the various tumour substrate morphological variants.

Table 2 presents the occurrence of various morphological variants in the tumour substrate identified in the plasmacytoma biopsy material taken from MM patients. Microscopic examination of bone plasmacytoma biopsy tissue from 10 patients revealed massive infiltration by mature plasma cells, and in 4 patients, there was massive infiltration by tumour cells with an immature morphology. Microscopic examination of extramedullary plasmacytoma biopsies revealed massive infiltration by mature plasma cells in 3 patients. In 4 cases, the tumour substrate of the plasmacytoma was represented by tumour cells with an immature morphology.

**Table 2:** The frequency of various plasmacytoma morphological variants in MM patients

Plasmacytoma localisation	Morphological plasmacytoma variant-massive infiltration by plasma cell with		p
	Mature morphology	Immature morphology	
Bone	71.4% (10/14)	28.6% (4/14)	0.35
Extramedullary	42.9% (3/7)	57.1% (4/7)	

When comparing the morphological patterns of bone and extramedullary plasmacytoma, no significant differences were revealed; however, the substrate of extramedullary plasmacytoma was more often represented by tumour cells with an immature morphology than was that of bone plasmacytoma (57.1% vs. 28.6%, respectively).

### Immunohistochemical study of plasmacytoma in MM patients

We carried out a comparative analysis of the quantitative parameters of the studied marker expression in the biopsy specimens of bone and extramedullary plasmacytomas. Table 3 presents the average values for marker expression.

**Table 3:** The parameters of marker’s expression in bone and extramedullary plasmacytoma in MM patients

Marker	MM patients with plasmacytoma		p
	Bone (***n*** = 14)	Extramedullary (***n*** = 7)	
	Expression parameters (%)		
	Мean ± SE		
CD56	54.29 **±** 10.18	28.57 **±** 14.86	0.165
CD166	36.29 **±** 7.61	9.57 **±** 8.46	**0.044**
CXCR4	45.50 **±** 9.35	31.71 **±** 13.91	0.413
Ki-67	25.36 **±** 5.63	61.43 **±** 10.50	**0.004**
c-MYC	33.29 **±** 8.37	37.86 **±** 10.57	0.748

When analysing the results obtained, a significant difference was found in terms of the cell adhesion molecule CD166 and the Ki-67 protein. The mean values of CD166 expression in cells of bone plasmacytoma were significantly higher (*p* = 0.044) than those of extramedullary plasmacytoma and amounted to 36.29 ± 7.61% versus 9.57 ± 8.46%, respectively. It was also demonstrated that the mean Ki-67 values in extramedullary plasmacytoma cells were significantly higher (*p* = 0.004) than those in bone plasmacytoma cells and amounted to 61.43 ± 10.50% versus 25.36 ± 5.63%, respectively.

We expected to detect low expression of the adhesion molecule CD56 and the chemokine receptor CXCR4 on plasmacytoma cells. According to a theory, by losing adhesion receptors, myeloma cells escape the control of the microenvironment and form tumours outside the bone marrow. However, according to our data, both bone plasmacytoma cells and extramedullary plasmacytoma cells could express these molecules. This is probably not the main mechanism of plasmacytoma pathogenesis. The c-MYC gene product is a transcription factor involved in cell proliferation, cycle regulation, and apoptosis. It seemed interesting to us to estimate the expression of the c-MYC protein in bone and extramedullary plasmacytoma cells. Differences in the expression of this protein depending on the type of plasmacytoma or cell morphology were not detected.

**Fig. 5:**
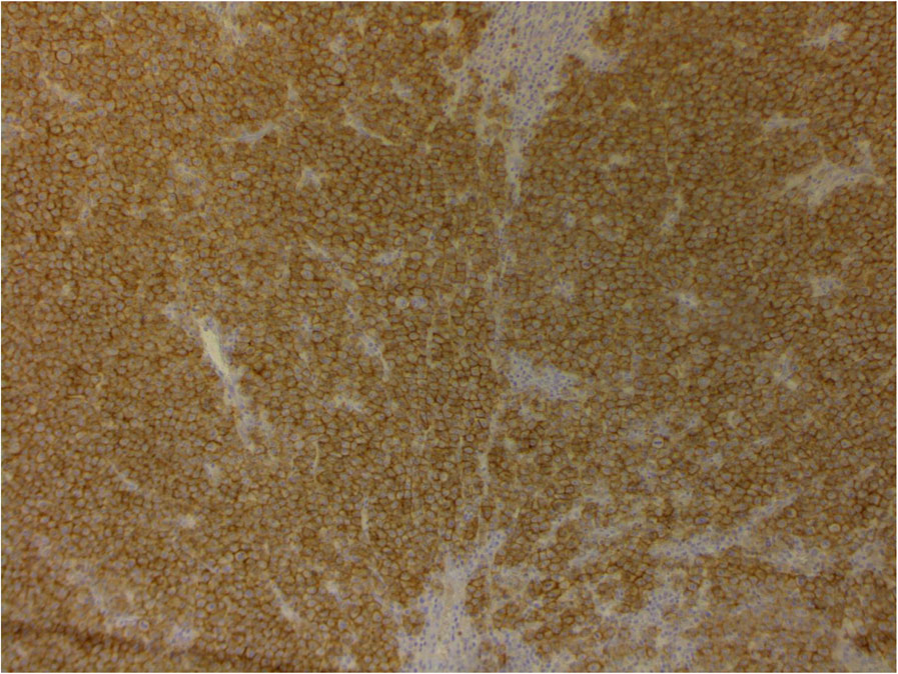
Mammary gland extramedullary plasmacytoma. Staining using antibodies to CD56, enzyme immunoassay. Membrane expression more than 90% of the tumor cells. × 200

**Fig. 6:**
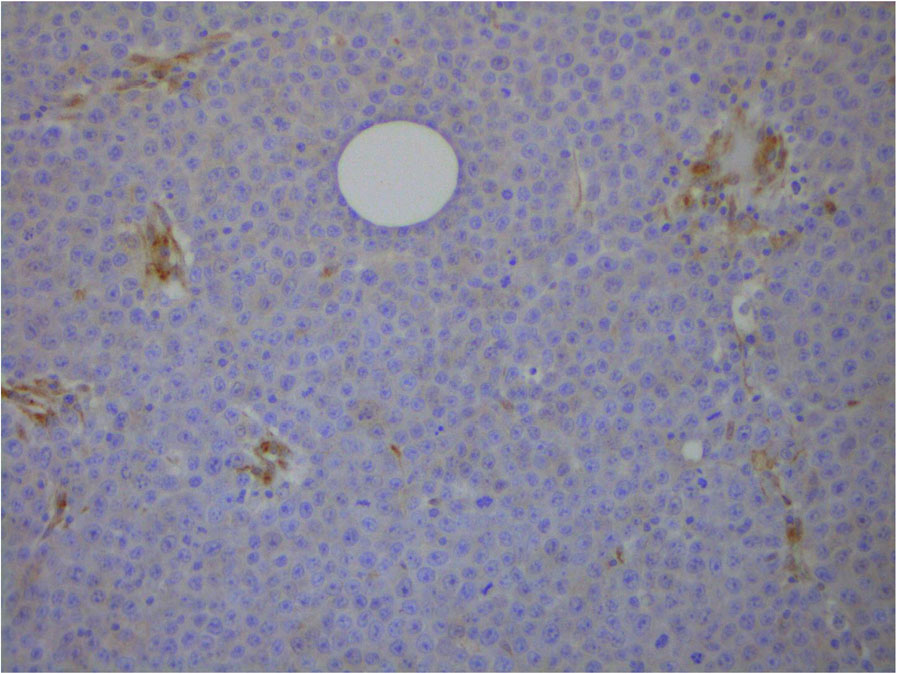
Mammary gland extramedullary plasmacytoma. Staining using antibodies to CD166, enzyme immunoassay. Cytoplasmic and membrane expression. Expression of CD166 in single cells. × 200

**Fig. 7:**
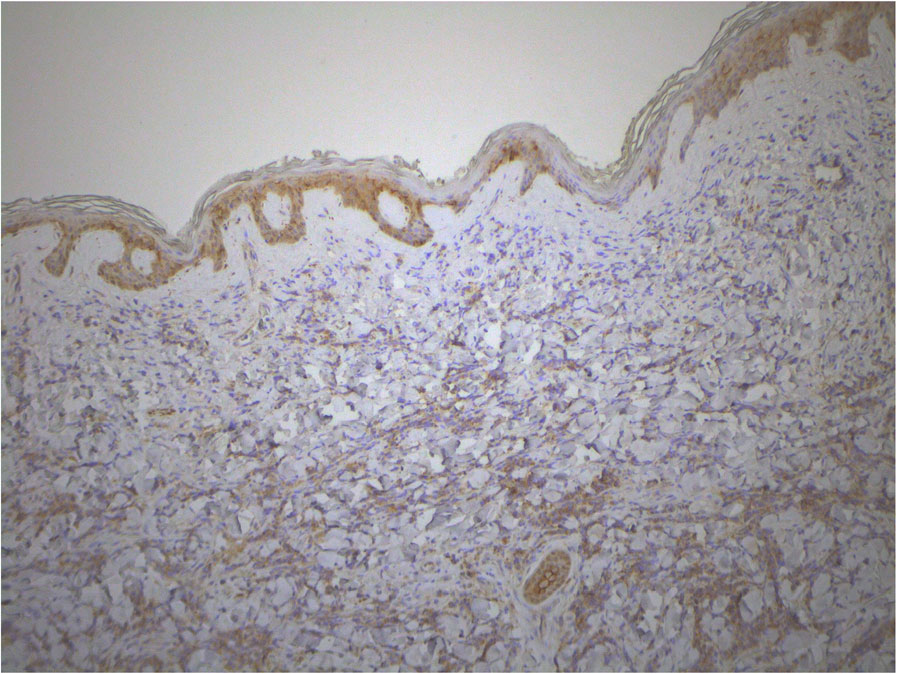
Skin extramedullary plasmacytoma. Staining using antibodies to CD166, enzyme immunoassay. Cytoplasmic and membrane expression in 30% of the tumor cells. × 100

**Fig. 8:**
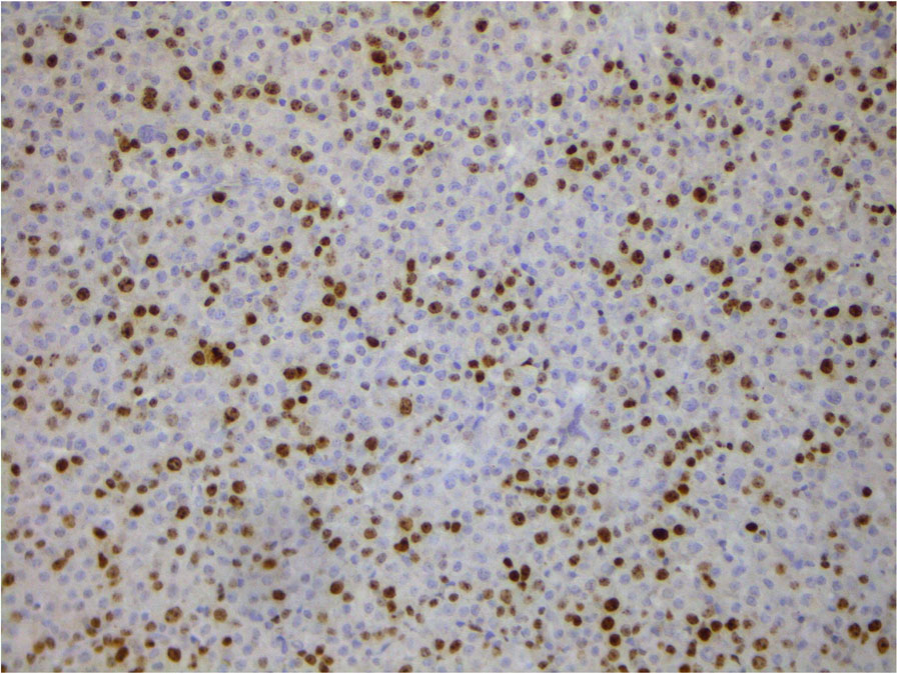
Rib bone plasmacytoma. Staining using antibodies to Ki-67. Nuclear expression in 25% of the tumor cells. × 200

**Fig. 9:**
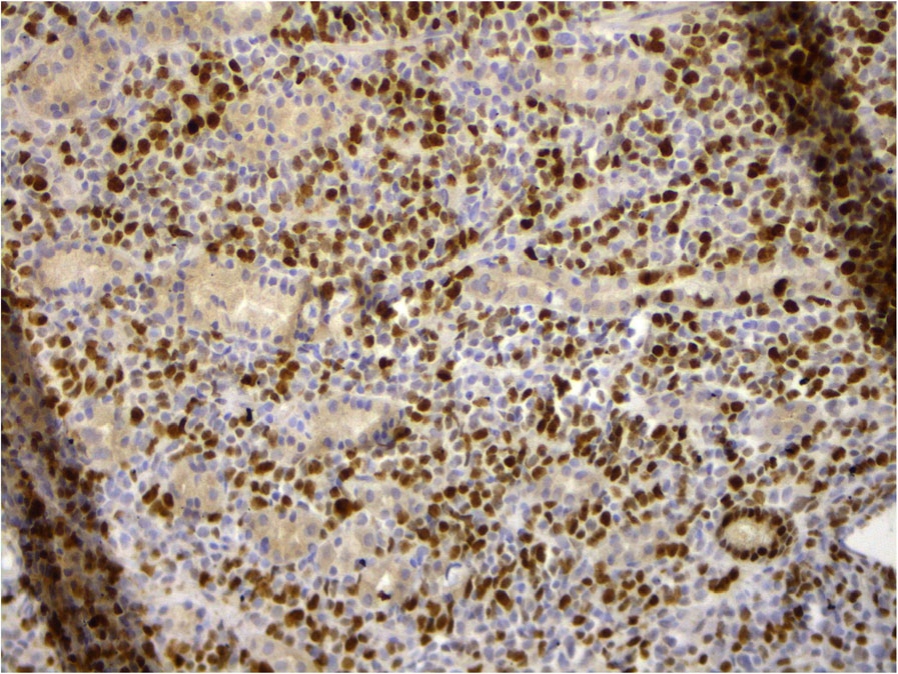
Stomach extramedullary plasmacytoma. Staining using antibodies to Ki-67. Nuclear expression in 65% of the tumor cells. × 200

**Fig. 10:**
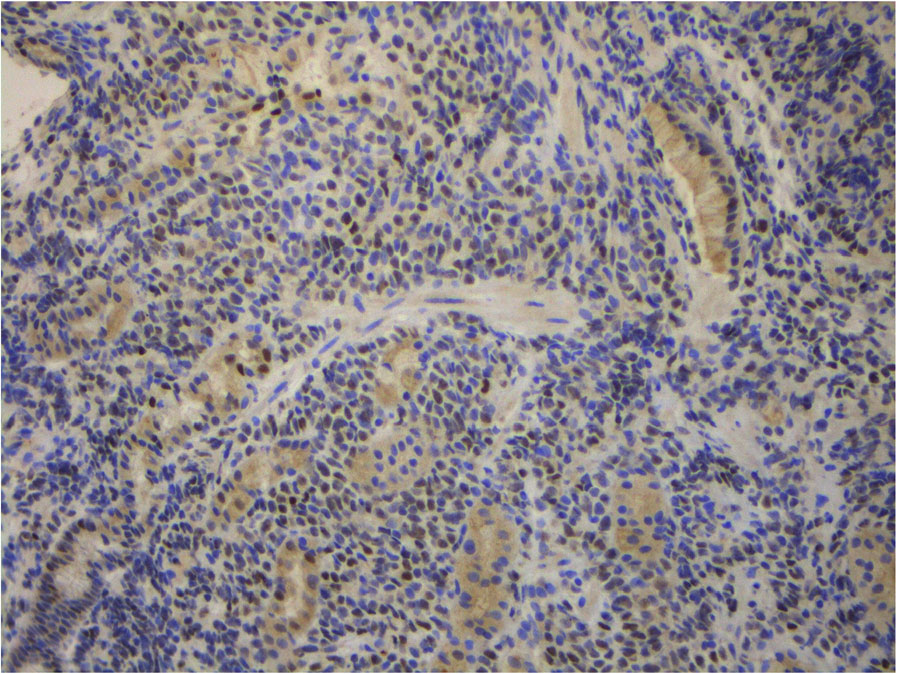
Stomach extramedullary plasmacytoma. Staining using antibodies to c-MYC. Nuclear expression in 15% of the tumor cells. × 200

Figures 5, 6, 7, 8, 9 and 10 present images of IHC-evaluated plasmacytoma specimens from MM patients.

### Correlation of marker expression in plasmacytoma cells with the morphological features of the plasmacytoma substrate

We analysed plasmacytoma cell expression of the studied markers depending on the plasmacytoma cell morphological structure. In 13 patients, the plasmacytoma tumour substrate was represented by mature plasma cells. In 8 patients, the cells were immature. Table 4 shows the mean average values for marker expression in the plasmacytomas with a mature or an immature cell morphology.

**Table 4:** Parameters of the marker’s expression in plasmacytoma specimens from patients with MM

Marker	Plasmacytoma cell morphology		p
	Mature (*n* = 13)	Immature (*n* = 8)	
	Expression parameters (%)		
	Мean ± SE		
CD56	39.23 **±** 9.28	56.25 **±** 17.10	0.351
CD166	38.23 **±** 8.37	9.75 **±** 5.87	**0.025**
CXCR4	33.85 **±** 8.47	52.38 **±** 14.65	0.253
Ki-67	29.77 **±** 7.73	49.75 **±** 9.61	0.124
c-MYC	34.15 **±** 7.15	35.88 **±** 13.06	0.901

When analysing the results obtained, a statistically significant difference was revealed only for the cell adhesion molecule CD166. The average values of CD166 expression in cells with a mature morphology were significantly higher than those in cells with an immature morphology (*p* = 0.025) and amounted to 38.23 ± 8.37% versus 9.75 ± 5.87%, respectively.

The pathobiological basis of the higher rates of CD166 expression in mature plasmacytoma plasma cells, in contrast to immature cells, is currently not fully understood and requires further study.

## Discussion

There have only been a few studies conducted to investigate the morphological features of plasmacytoma biopsies. Some studies have shown that cells in bone plasmacytoma are more often characterized by a mature morphology, while cells in extramedullary plasmacytoma are represented by young, immature cell types [9–11]. It should be noted that most of these works are devoted to the study of extramedullary MM relapses. However, importantly, the morphological features of tumour substrate cells in newly diagnosed MM and relapsed disease may differ.

We investigated the morphological features of the plasmacytoma substrate in 14 patients with bone plasmacytoma and 7 patients with extramedullary plasmacytoma among newly diagnosed MM patients. The small sample size was due to the rare rate of plasmacytoma, especially extramedullary foci. Moreover, biopsying for plasmacytoma is not a compulsory method for MM diagnosis. If we have irrefutable proof of diagnosis (CRAB syndrome or infiltration of plasma cells into the bone marrow), we need to start treatment without biopsying for plasmacytoma. However, sometimes the diagnosis of MM is established after tumour biopsy. This group of patients is presented in our study. When comparing the morphological features of bone and extramedullary plasmacytoma, no significant differences were revealed; however, the substrate of extramedullary plasmacytoma was more often represented by tumour cells with an immature morphology than was the bone plasmacytoma substrate (57.1% vs. 28.6%, respectively).

A possible mechanism for tumour plasma cell expansion beyond the bone marrow is the downregulation of adhesion molecule expression (for example, CD56), which results in cells losing their connection with the stromal microenvironment [18, 19]. The role of the expression of another cell adhesion molecule, CD166, is now being actively investigated in MM. A study on mouse models showed that CD166 blocked osteoblastogenesis by inhibiting RUNX2 expression, which is an important transcription factor influencing the differentiation of osteoblasts. In addition, CD166 activated osteoclastogenesis, shifting the balance between RANKL and osteoprotegerin. CD166 blockade in mouse myeloma cells resulted in longer survival, a lower total tumour mass, and less pronounced osteolysis than in mouse with CD166-positive cells [20]. Thus, it is assumed that CD166 is a predictor for lytic bone lesions, as it participates in osteogenic modulation.

In our study, a statistically significant difference in the expression of CD166 was revealed after comparing the expression of this marker in bone and extramedullary plasmacytoma cells. The mean values of CD166 expression in bone plasmacytoma cells were significantly higher than those in the extramedullary plasmacytoma cells (*p* = 0.033) and amounted to 36.29 ± 7.61% versus 9.57 ± 8.46%, respectively. This may indicate the involvement of CD166 in the mechanisms of bone destruction. In addition, it was demonstrated that the mean expression values of CD166 in plasmacytoma cells with a mature morphology were significantly higher than those in plasmacytoma cells with an immature morphology (*p* = 0.012) and amounted to 38.23 ± 8.37% versus 9.75 ± 5.87%, respectively.

SDF-1α and a chemokine receptor, CXCR4, are involved in the processes of cell homing and cell migration. It has been indicated that myeloma cells can express these markers on their surface [21, 22]. Downregulation of chemokine receptor expression is considered to be a possible mechanism leading to a weakening of the connection between myeloma cells and the bone marrow stroma, thus promoting myeloma cell dissemination. The absence of CXCR4 on the tumour plasma cell surface, while not being a strict predictive factor of extramedullary lesions, probably plays a role in the formation of extraosseous foci.

There have been only a few studies on the roles of chemokine receptors in MM pathogenesis, and the data presented in the literature are contradictory. We demonstrated a high frequency of CXCR4 expression in plasmacytoma cells. These results are comparable with data from a study by M. Weinstock et al., where 38.5% of patients showed CXCR4 expression in the tumour substrate [23].

According to the data presented in the literature, the proliferative activity of tumour cells in MM is low. An increased Ki-67 index is a marker of active cell growth and correlates with the progression of the disease. Researchers at the Mayo Clinic showed that a high level of proliferative activity, even with a minimum number of plasma cells in the bone marrow, is a risk factor for early relapse and high mortality [24].

We noticed that in extramedullary plasmacytoma cells, there were higher values for the Ki-67 index observed in comparison with bone plasmacytoma cells. These data are comparable with the results of a single-centre study from Germany, which demonstrated high proliferative activity in extramedullary plasmacytoma biopsy specimens from 24 patients with extramedullary MM relapse [25]. Interestingly, our study shows that Ki67 expression is higher in extramedullary plasmacytoma than in bone plasmacytoma regardless of cell morphology. There has been an insufficient number of studies devoted to the investigation of c-MYC in the tumour substrate of plasmacytoma. In a study by L. Billecke et al., overexpression of the c-MYC gene in the plasmacytoma substrate was observed in 18% of patients with bone plasmacytoma and 28% of patients with extramedullary plasmacytoma [26].

There have been no studies of plasmacytoma substrates in large patient populations.

This is partly because extramedullary lesions in MM are rare and it is not always possible to perform a plasmacytoma biopsy due to its inaccessible localization.

## Conclusion

There are several presumptive mechanisms that contribute to the extramedullary spread of clonal plasma cells in MM (for example, downregulation of adhesion molecule and chemokine receptor expression and angiogenesis upregulation). In the literature, some researchers analysed the expression of CD56 in plasmacytoma cells in a small study, while other authors studied the expression of chemokine receptors on tumour cells [1, 3]. The uniqueness of our work is that we studied the plasmacytoma cell expression of a number of IHC parameters that are important for understanding why myeloma cells “escape” bone marrow control. In addition, we compared the expression values in bone and extramedullary plasmacytoma cells to search for differences. Even in such a small sample, some differences in expression could be identified, which may indicate that different mechanisms lead to the formation of bone and extramedullary plasmacytoma. Specifically, the expression of a cell adhesion molecule, CD166, in extramedullary plasmacytoma cells was almost 4 times lower than that in bone plasmacytoma cells, which may indicate the involvement of CD166 in osteogenic modulation. We also analysed the Ki-67 index, and we showed the high proliferative activity of extramedullary plasmacytoma cells at the time of MM diagnosis.

A more thorough study of the tumour substrate in MM is likely to reveal some regular patterns in the formation of extramedullary lesions, which will contribute to a better understanding of the tumour’s biology.

## Data Availability

All data analysed during this study are included in this published article and its supplementary information files.

## Abbreviations

MM: Multiple myeloma
IHC: Immunohistochemistry
